# Vitamin D moderates the interaction between 5-HTTLPR and childhood abuse in depressive disorders

**DOI:** 10.1038/s41598-020-79388-7

**Published:** 2020-12-28

**Authors:** Sarah Bonk, Johannes Hertel, Helena U. Zacharias, Jan Terock, Deborah Janowitz, Georg Homuth, Matthias Nauck, Henry Völzke, Henriette Meyer zu Schwabedissen, Sandra Van der Auwera, Hans Jörgen Grabe

**Affiliations:** 1 Department of Psychiatry and Psychotherapy, University Medicine Greifswald, Ellernholzstraße 1-2, 17475 Greifswald, Germany; 2 Molecular Systems Physiology Group, National University of Ireland, Galway, Ireland; 3 Department of Psychiatry and Psychotherapy, HELIOS Klinikum Stralsund, Stralsund, Germany; 4 Interfaculty Institute for Genetics and Functional Genomics, University Medicine Greifswald, Greifswald, Germany; 5 Institute of Clinical Chemistry and Laboratory Medicine, University Medicine Greifswald, Greifswald, Germany; 6 DZHK (German Centre for Cardiovascular Research), University Medicine, Partner Site Greifswald, Greifswald, Germany; 7 Institute for Community Medicine, University Medicine Greifswald, Greifswald, Germany; 8 Department Pharmaceutical Sciences, University of Basel, Basel, Switzerland; 9 German Center for Neurodegenerative Diseases DZNE, Site Rostock/Greifswald, Germany

**Keywords:** Depression, Biomarkers, Statistical methods, Genetics, Risk factors

## Abstract

A complex interplay between genetic and environmental factors determines the individual risk of depressive disorders. Vitamin D has been shown to stimulate the expression of the tryptophan hydroxylase 2 (TPH2) gene, which is the rate-limiting enzyme for serotonin production in the brain. Therefore, we investigate the hypothesis that serum vitamin D levels moderate the interaction between the serotonin transporter promotor gene polymorphism (5-HTTLPR) and childhood abuse in depressive disorders. Two independent samples from the Study of Health in Pomerania (SHIP-LEGEND: *n* = 1 997; SHIP-TREND-0: *n* = 2 939) were used. Depressive disorders were assessed using questionnaires (BDI-II, PHQ-9) and interview procedures (DSM-IV). Besides serum vitamin D levels (25(OH)D), a functional polymorphism (rs4588) of the vitamin D-binding protein is used as a proxy for 25(OH)D. S-allele carriers with childhood abuse and low 25(OH)D levels have a higher mean BDI-II score (13.25) than those with a higher 25(OH)D level (9.56), which was not observed in abused LL-carriers. This significant three-way interaction was replicated in individuals with lifetime major depressive disorders when using the rs4588 instead of 25(OH)D (*p* = 0.0076 in the combined sample). We conclude that vitamin D relevantly moderates the interaction between childhood abuse and the serotonergic system, thereby impacting vulnerability to depressive disorders.

## Introduction

Major depressive disorders (MDD) have an estimated heritability of only 31–42%, clearly indicating additional contributions by environmental factors to MDD^1^.

In 2003, Caspi et al. reported an association between the S allele of the 5-HTTLPR serotonin transporter promoter region with an increased risk of depression in individuals who where exposed to stressful life events^2^. This work stimulated further research on this topic, but yielded ambiguous results^3–6^.

Recently, Border et al*.* investigated the role of 18 historical candidate genes and the gene-by-interaction hypotheses for depressive disorders in UK Biobankand Psychiatric Genomics Consortium samples^7^. They found no support for 5-HTTLPR as the primary effect, nor for its interaction with childhood abuse.

This result raises questions about current views on the biological role of gene polymorphisms in complex mental disorders. However, to date, only simple models of direct gene effects or two-way gene-environment interactions have constituted the primary research focus. However, biological interactions and pathways might be numerous, and an individual’s settings and life history will be highly complex^8^. The challenge is to define plausible hypotheses based on complex models that are still testable under an accepted statistical approach.

Following this line of research on the putative complex interaction effects of 5-HTTLPR, vitamin D may constitute an interesting and important moderator of the 5-HTTLPR-driven gene × environment interaction. Vitamin D is well-known for its wide range of physiological effects with impacts on both serotonin metabolism and depression^9–11^. Vitamin D stimulates the expression of the rate-limiting enzyme tryptophan hydroxylase 2 (TPH2) gene in human and rat brain cells^12^, and it thereby contributes to serotonin biosynthesis. Likewise, the association between vitamin D and depressive disorders was repeatedly confirmed even after adjusting for lifestyle, sociodemographic and metabolic factors^13–15^. The evidence suggested that low vitamin D levels affect current depressive mood states but not depression as a trait^16^.

One of the strongest predictors for depression are traumata in general and especially childhood traumata, which can increase the risk of depression by several times^17^. The prevalence is at least 10% for abuse and 20% for neglect. These rates might be much higher in at-risk populations^18^. Many further studies have investigated gene × environment interactions in relation to childhood trauma and depression^19–21^. A particularly interesting suggestion from individual studies is that the role of genes in depression is more strongly pronounced for traumata occurring during psychological developmental phases such as childhood and youth.

Here, we examined the hypothesis of a 5-HTTLPR-gene × childhood abuse interaction model for depression, i.e., current depressive symptoms or MDD lifetime, that is relevantly moderated by the vitamin D serum levels (25-hydroxyvitamin D (25(OH)D)) in population-based cohort SHIP-TREND-0 from the Study of Health in Pomerania (SHIP).

To validate our results, we employed vitamin D metabolism affecting a functional polymorphism, SNP rs4588, as proxy in a second independent data set (SHIP-LEGEND). This SNP was the top hit in a genome-wide association study on serum 25-(OH)D and is located in the vitamin D binding protein (DBP) gene. Polymorphisms of DBP can affect the 25(OH)D levels on a similar scale as vitamin D intake, calcium intake and body-mass-index (BMI)^22^.

Thus, the effect captured by rs4588 acts independently from other possibly unmeasured environmental or biological factors, and it is not biased by measurement inaccuracies of vitamin D serum levels^22–24^.

We further hypothesize that the “state” variable 25(OH)D (serum level) would be associated with current depressive symptoms, whereas the “trait” variable rs4588 would impact the risk of lifetime depression in interactions with childhood abuse and 5-HTTLPR.

## Materials and methods

### Sampling and phenotyping methods

The investigations in both studies were performed in accordance with the Declaration of Helsinki, including the written informed consent of all participants. The survey and study methods were approved by the institutional review boards of the University of Greifswald.

Further information about the sampling, Vitamin D (Fig. S1), phenotyping and genotyping methods is given in the Supplement.

#### Sample and sample recruitment

Data from the Study of Health in Pomerania (SHIP) were used^25–27^. The target population was comprised of adult German (Caucasian) residents in north-eastern Germany. From the 4 308 participants of the baseline SHIP-0 (1997–2001), 2 400 continued to participate in the follow-up “Life-Events and Gene-Environment Interaction in Depression” (SHIP-LEGEND, 2007–2010).

In 2008, an independent new sample (SHIP–TREND-0) with 4 420 subjects from the same area was drawn, and examinations similar to those performed for the SHIP-0 were undertaken.

We excluded subjects from both cohorts who were missing information on childhood abuse, genetic polymorphisms (5-HTTLPR, rs25531 or rs4588), MDD or BDI-II/PHQ-9. The remaining sample comprised *n* = 1 997 subjects for SHIP-LEGEND and *n* = 2 939 subjects for SHIP-TREND-0 with vitamin D (25(OH)D)-based analyses; n = 2 901 subjects were available in the SHIP-TREND-0.

#### Phenotype measures

In SHIP-LEGEND and SHIP-TREND-0, a diagnostic interview on mental disorders was performed based on the Diagnostic and Statistical Manual for Mental Disorders (IV edition) diagnostic criteria^27,28^. Additional psychometric assessments included the Beck depression inventory (BDI-II, SHIP-LEGEND), patient health questionnaire (PHQ-9, SHIP-TREND-0), and childhood trauma questionnaire (CTQ, SHIP-LEGEND and SHIP-TREND-0). The BDI-II measures current depressive symptoms with high reliability and validity using a 21-item self-report questionnaire^29^. The PHQ-9 is a 9-item self-report questionnaire that also has high reliability and validity^30^.

The PHQ-9 score was transformed into the BDI-II according to Wahl et al*.*^31^ to create one common variable on depressive symptoms for both cohorts.

Our PHQ-9 scores for SHIP-TREND-0 and the transformed BDI-II values were consistent with a correlation of > 0.9.

The CTQ was used for self-reporting of childhood maltreatment, including emotional, physical and sexual abuse^17,32,33^. It comprises 34 items that are rated on a five-point Likert scale with higher scores indicating more self-rated exposure to traumatic events. In addition to a dimensional scoring procedure, the following threshold scores were used to determine the severity of abuse: none = 0, mild = 1, moderate = 2, and severe to extreme = 3. To investigate the role of increasingly severe of childhood abuse in G × E interactions, we generated a dichotomized variable of overall abuse. A subject was rated as positive for overall abuse when at least one of the abuse sub-dimensions received a severity score of at least mild.

#### Vitamin D measurement

Venous blood samples were taken in the SHIP-TREND-0 from the cubital vein of the participants in the supine position. The samples were taken throughout the year and stored at -80 °C. Their serum 25(OH)D concentrations were determined on an IDS-iSYS Multi-Discipline Automated Analyser (Immunodiagnostic Systems Limited, Frankfurt am Main, Germany).

For the regression analysis, the continuous 25(OH)D serum levels were divided into four quantiles, with the quartile containing the lowest 25(OH)D serum levels (17.2 ng/ml) considered as an at risk group against the remaining sample.

### Genetic methods

#### Genotyping of the 5-HTTLPR

The SLC6A4 gene harbours a variable number tandem repeat (VNTR) polymorphism in the transcription control region of the gene. Both variants (Short, Long) differ by a 43-bp insertion/deletion (“biallelic” 25-HTTLPR). Within the inserted fragment, an additional common single nucleotide polymorphism (SNP) occurs (rs25531). This finding suggested that 5-HTTLPR is triallelic, with S-, L_A_- and L_G_ -alleles.

Based on previous reports about gene expression, we classified the genotypes into three functional ‘‘triallelic’’ genotypes: L_A_L_A_ = LL; L_G_L_A_ or SL_A_ = SL; and L_G_L_G_ or L_G_S or SS = SS^34^. However, recently, the functional relevance of rs25531 has been called into question^35^. Even so, we report the results of the three-way interaction for this triallelic 5-HTTLPR. While SS and SL are considered separately in the descriptive statistics, they are grouped together for the regression^25^.

#### Genotyping of rs4588

The SHIP-0 sample (*n* = 4 070) was genotyped using the Affymetrix Human SNP Array 6.0. Genotyping in SHIP-TREND-0 was performed using the Illumina HumanOmni 2.5-Quad (*n* = 986) and the Illumina GSA-24 (*n* = 3 133).

Genotype imputation was performed using the HRCv1.1 reference panel.

The SNP was imputed with an imputation quality of > 0.99 in all three batches.

### Statistical analyses

The final case numbers were 1997 for SHIP-LEGEND and 2 939 for SHIP-TREND-0. Vitamin D (25(OH)D) was available in *n* = 2 901 subjects of SHIP-TREND-0. Missing values were not imputed.

The seasonal variation of the 25(OH)D serum levels is demonstrated in the Supplement.

#### 25(OH)D moderated two-way interaction regression on BDI-II and MDD in SHIP-TREND-0

Linear and logistic regressions with robust estimates and robust standard errors were applied to investigate the association between 25(OH)D, 5-HTTLPR (rs25531), and childhood abuse with regard to BDI-II and MDD in SHIP-TREND-0. The primary focus of the analysis was on the three-way interaction (25(OH)D × 5-HTTLPR × childhood abuse). However, all possible direct associations as well as two-way interactions (25(OH)D × 5-HTTLPR, 25(OH)D × childhood abuse, 5-HTTLPR × childhood abuse) were analysed for informative reasons.

#### rs4588 as Proxy for 25(OH)D

We tested whether the SNP rs4588 was a suitable marker for 25(OH)D serum levels in SHIP-TREND-0 and used rs4588 as a proxy for 25(OH)D serum levels in SHIP-LEGEND and SHIP-TREND-0. Again, the three-way interaction as well as the corresponding direct association and two-way interaction models among rs4588, 5-HTTLPR and childhood abuse were calculated with lifetime MDD as the outcome. Corresponding analyses were performed with rs4588 in the combined sample (SHIP-TREND-0 + SHIP-LEGEND).

The same analyses as those used for MDD were performed for BDI-II as the outcome in SHIP-LEGEND, SHIP-TREND-0, and the combined sample.

All the regression models were adjusted for participant age, sex, genetic principal components (PC1-3), batch effects for the different GWAS chips and study population in the combined sample analyses.

The analyses with 25(OH)D were additionally adjusted for the BMI, physical activity, smoking (non, ex- *vs* current smoker) and season. The seasonal variations in the 25(OH)D serum levels were accounted for by the metric variable “day in the year (1–365)” using restricted cubic splines with four equally spaced knots. The same approach was used for age adjustment to account for non-linear age effects on depression severity. Restricted cubic splines have been considered to be superior to categorized age groups^36^.

Statistical analyses were performed using *R* software version 3.4.4^37^.

## Results

The sample characteristics are given in Table 1. The SNP rs4588 as well as the triallelic 5-HTTLPR genotypes were in accordance with Hardy–Weinberg equilibrium for SHIP-LEGEND and SHIP-TREND-0.

**Table 1:** Summary statistics of SHIP-LEGEND and SHIP-TREND by sex.

Variable	SHIP-LEGEND (n = 1 997)			SHIP-TREND-0 (n = 2 939)			Combined sample (n = 4 936)			
	Males (n = 957)	Females (n = 1 040)	Comparison males vs females	Males (n = 1 447)	Females (n = 1 492)	Comparison males vs females	Males (n = 2 404)	Females (n = 2 532)	Comparison males vs females	Comparison SHIP-LEGEND vs SHIP-TREND-0
Age, years (mean(sd))	56.8 (14.1)	54.1 (13.3)	t = 4.5, *p* < 0.001	52.3 (15.3)	50.8 (14.9)	t = 2.8, * p* = 0.005	54.1 (15.0)	52.2 (14.4)	t = 4.7, * p* < 0.001	t = 9.1, *p* < 0.001
MDD lifetime (N(%))	119 (12.4%)	253 (24.3%)	*χ*^*2*^ = 46.5 * p* < 0.001	195 (13.5%)	338 (22.7%)	*χ*^*2*^ = 41.7, * p* < 0.001	314 (13.1%)	591 (22.8%)	*χ*^*2*^ = 87.0, * p* < 0.001	*χ*^*2*^ = 0.2, *p* = 0.66
BDI-II score (mean(sd))	5.7 (6.5)	7.2 (8.0)	t = − 4.5, * p* < 0.001	7.2 (5.4)	8.3 (5.6)	t = − 5.5, * p* < 0.001	6.6 (5.9)	7.9 (6.7)	t = − 6.9, * p* < 0.001	t = − 7.2, * p* < 0.001
PHQ-9 score (mean(sd))	NA	NA	NA	12.4 (3.5)	13.3 (3.6)	t = − 6.4, * p* < 0.001	NA	NA	NA	NA
Abuse^†^ (N(%))	152 (15.9%)	206 (19.9%)	*χ*^*2*^ = 5.2, * p* = 0.022	209 (14.4%)	282 (18.9%)	*χ*^*2*^ = 10.5, * p* = 0.001	361 (15.0%)	488 (19.3%)	*χ*^*2*^ = 15.7, * p* < 0.001	*χ*^*2*^ = 1.2, *p* = 0.27
**rs4588 (N(%))**										
CC	478 (50%)	520 (50%)	*χ*^*2*^ = 0.02, * p* = 0.99	704 (49%)	723 (48%)	*χ*^*2*^ = 0.05, * p* = 0.97	1 182 (49%)	1 243 (49%)	*χ*^*2*^ = 0.01, * p* = 0.99	*χ*^*2*^ = 1.0, * p* = 0.61
CA	401 (42%)	437 (42%)		627 (43%)	646 (43%)		1 028 (43%)	1 083 (43%)		
AA	78 (8%)	83 (8%)		116 (8%)	123 (9%)		194 (8%)	206 (8%)		
**5-HTTLPR (N(%))**										
SS	195 (20%)	212 (20%)	*χ*^*2*^ = 0.89, * p* = 0.64	305 (21%)	337 (23%)	*χ*^*2*^ = 0.98, * p* = 0.61	500 (21%)	549 (22%)	*χ*^*2*^ = 0.93, * p* = 0.63	*χ*^*2*^ = 1.5, * p* = 0.47
SL	475 (50%)	535 (51%)		725 (50%)	733 (49%)		1 200 (50%)	1 268 (50%)		
LL	287 (30%)	293 (29%)		417 (29%)	422 (28%)		704 (29%)	715 (28%)		
Vitamin D level (mean(sd))	NA	NA	NA	24.5 (9.3)	24.4 (10.0)	t = 0.19, *p* = 0.85	NA	NA	NA	NA

NA, not available in the cohort.

^†^None versus at least mild abus, *t*-test for metric data, *χ*^*2*^-test for non-metric data.

Significant differences between the subjects of SHIP-LEGEND and SHIP-TREND-0 were apparent for age (*p* < 0.001) and depression score (*p* < 0.001). The age differences are caused by the different survey waves of SHIP-LEGEND (second follow-up of SHIP-0) and SHIP-TREND-0 (new baseline cohort).

Due to the low minor allele frequency in rs4588 and the small number of homozygous AA carriers, we summarized AA/AC versus CC in the regression analyses.

### Vitamin D moderates the two-way interaction of childhood abuse and 5-HTTLPR on the BDI-II score

**Figure 1:**
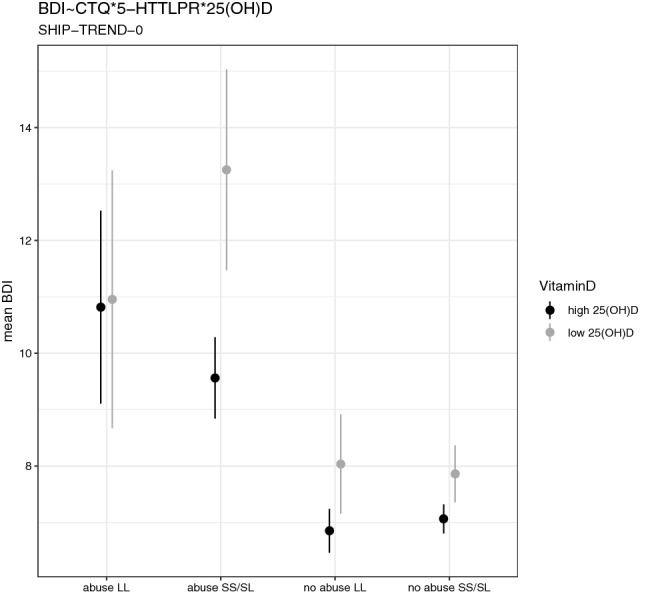
Mean and confidence intervals in depression symptoms (BDI-II) for different subgroups of the three-way interaction childhood trauma questionnaire (CTQ, abuse vs. no abuse), 5-HTTLPR (LL vs. SL/SS) and vitamin D (25(OH)D high ≥ 17.2 ng/ml vs. low < 17.2 ng/ml) in SHIP-TREND-0.

We found a significant three-way interaction effect of childhood abuse, 25(OH)D serum levels and the 5-HTTLPR genotype on the BDI-II score in SHIP-TREND-0 (*β* = − 3.55 (− 7.00 – − 0.10 95% CI), *p* = 0.043). The mean BDI-II scores in the individual groups emerging from this interaction are shown in Fig. 1, and all the *p*- and *β-* values are summarized in Table 2.

**Table 2:** Linear regression of BDI-II scores and different primary effects with robust estimates adjusted for age and sex in SHIP-TREND-0. For the effects including the 25(OH)D, the regression has additionally been adjusted for the body mass index, physical activity, smoking, and season.

Outcome: BDI-II	SHIP-TREND-0	
	*β* (95% CI) coefficient	*p*-value
**Primary effect**		
Abuse	3.22 (2.56–3.89)	< 2.2e−16
5-HTTLPR	− 0.05 (− 0.50–0.40)	0.831
25(OH)D serum levels	1.32 (0.80–1.85)	9e-07
**Two-way interaction**		
Abuse*5-HTTLPR	0.52 (− 1.07–2.11)	0.522
Abuse*25(OH)D	1.62 (0.01–3.22)	0.049
5-HTTLPR*25(OH)D	− 0.23 (− 1.32–0.86)	0.68
**Three-way interaction**		
Abuse*5-HTTLPR*25(OH)D	− 3.55 (− 7.00–0.10)	0.043

CI, confidence interval

The highest BDI-II scores were observed in the group with childhood abuse, low 25(OH)D serum levels, and 5-HTTLPR genotypes SL/SS. In case of childhood abuse and 5-HTTLPR genotype LL, the 25(OH)D level did not influence the mean BDI-II scores.

For additional analyses, we calculated all the associated primary and two-way interaction effects, see Table 2. No direct effect of the 5-HTTLPR genotype on BDI-II was found in SHIP-TREND-0, but childhood abuse and the 25(OH)D serum levels showed significant primary effects (childhood abuse: *β* = 3.22 (2.56 – 3.89 95% CI), *p* < 2.2e-16, 25(OH)D serum levels: *β* = 1.32 (0.80 – 1.85 95% CI), *p* < 9e-07).

In considering two-way interactions, only the interaction between childhood abuse and 25(OH)D was significant (*β* = 1.62 (0.01 – 3.22 95% CI), *p* < 0.049). No other significant two-way interaction effects on the BDI-II score were found.

All p-values and β values for the primary effects (childhood abuse, 5-HTTLPR and 25(OH)D serum levels) and their interactions on BDI-II are summarized in Table 2.

The only significant factor in the logistic regression on MDD using the 25(OH)D serum levels in SHIP-TREND-0 was childhood abuse (odds ratio (OR) = 2.50 (1.99 – 3.13 95% CI), *p* = 2.12e-15). No other factor or interaction was significant. All the *p*- and OR-values for the primary effects (childhood abuse, 5-HTTLPR and 25(OH)D serum levels) and their interactions on MDD are given in Supplementary Table S1.

### Direct and interaction effects on lifetime depression in SHIP-LEGEND and SHIP-TREND-0 using rs4588 as a proxy for the vitamin D levels

We used rs4588 as a proxy for the 25(OH)D serum levels in SHIP-LEGEND as an independent validation of the 25(OH)D serum level results in SHIP-TREND-0. Moreover, we also performed the rs4588-based analyses in SHIP-TREND-0 for further replication of this SNP effect. The rs4588 significantly predicted the 25(OH)D serum levels in SHIP-TREND-0 (*p* = 0.00719, *R*^*2*^ = 0.0025) in accordance with the literature^22–24,38^(see Supplementary Fig. S2).

All the *p*- and OR-values for the primary effects (childhood abuse, 5-HTTLPR, and rs4588) and their interactions on MDD are summarized in Table 3.

**Table 3:** Logistic regression of MDD values and different primary effects with robust estimates adjusted for age, sex and study cohort in SHIP-LEGEND, SHIP-TREND-0, and the combined sample (SHIP-LEGEND + SHIP-TREND-0).

Outcome: MDD	SHIP-LEGEND		SHIP-TREND-0		Combined	
	OR (95% CI)	*p* value	OR (95% CI)	*p* value	OR (95% CI)	*p* value
**Primary effect**						
Abuse	2.66 (2.04–3.46)	3.5e−13	2.50 (2.00–3.13	1.2e-15	2.57 (2.15–3.02)	< 2e-16
5-HTTLPR	0.97 (0.75–1.24)	0.793	1.01 (0.82–1.24)	0.932	0.99 (0.84–1.16)	0.889
rs4588	1.00 (0.79–1.25)	0.973	0.89 (0.74–1.08)	0.238	0.93 (0.81–1.08)	0.344
**Two-way interaction**						
Abuse*5-HTTLPR	1.16 (0.65–2.07)	0.614	1.03 (0.62–1.69)	0.920	1.10 (0.75–1.60)	0.636
Abuse*rs4588	1.49 (0.88–2.53)	0.142	0.78 (0.50–1.22)	0.281	1.04 (0.74–1.46)	0.836
5-HTTLPR*rs4588	0.67 (0.40–1.11)	0.12	0.83 (0.54–1.27)	0.390	0.75 (0.54–1.04)	0.089
**Three-way interaction**						
Abuse*5-HTTLPR*rs4588	0.26 (0.08–0.84)	0.024	0.41 (0.15–1.13)	0.088	0.35 (0.16–0.76)	0.0076

OR, odds ratio; CI, confidence interval.

#### rs4588 moderates the two-way interaction on MDD

We found a significant gene × gene × environment interaction effect on the lifetime MDD in SHIP-LEGEND (OR = 0.26 (0.08 – 0.84 95% CI), *p* = 0.024). This finding was replicated in SHIP-TREND-0 (OR = 0.41 (0.15 – 1.13 95% CI), *p* = 0.088) with a one-sided statistical significance. In the combined data set, the three-way-interaction yielded an OR of 0.35 (0.16 – 0.76 95% CI, *p* = 0.0076), as shown in Table 3.

**Figure 2:**
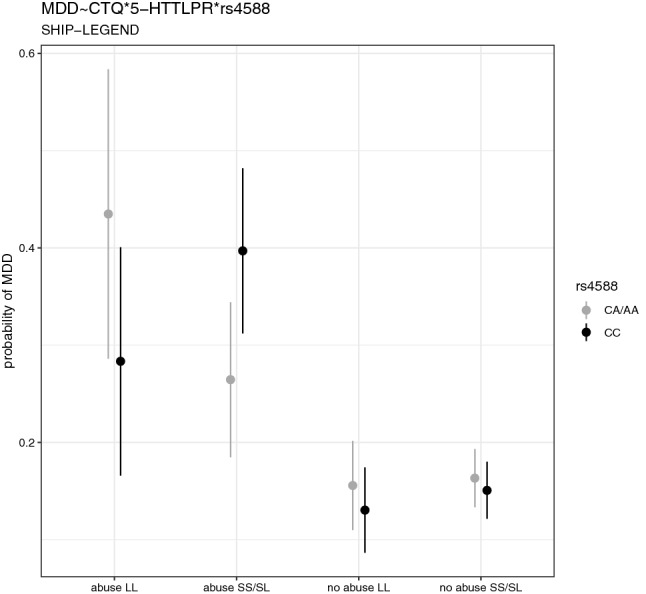
Mean and confidence intervals for probabilities of lifetime depression (MDD) for different subgroups of the three-way interaction childhood trauma questionnaire (CTQ, abuse vs. no abuse), 5-HTTLPR (LL vs. SL/SS) and rs4588 (CC vs. CA/AA) in SHIP-LEGEND.

The estimated probabilities of lifetime MDD in this three-way interaction in SHIP-LEGEND are shown in Fig. 2. The lowest probabilities are obtained for combinations with no childhood abuse, exhibiting only minor differences between the individual genotypes of rs4588 and 5-HTTLPR. The significant interaction effect was driven by opposite effects of the rs4588 genotypes depending on the 5-HTTLPR LL versus SL/SS genotypes in subjects with childhood abuse.

The three-way interaction on MDD in SHIP-TREND-0 and the combined sample are depicted in Supplementary Fig. S3 and S4.

We additionally calculated all the corresponding primary and two-way interaction effects. We found no direct effect of either the rs4588 or the 5-HTTLPR genotypes on lifetime depression in SHIP-LEGEND and SHIP-TREND-0, respectively. Again, childhood abuse was a major predictor of lifetime MDD in both SHIP cohorts (SHIP-LEGEND: OR = 2.66 (2.04 – 3.46 95% CI), *p* = 3.5e-13; SHIP-TREND-0: OR = 2.50 (2.00 – 3.13 95% CI), *p* = 1.2e-15; and combined: OR = 2.57 (2.15 – 3.02 95% CI), *p* < 2e-16).

There were no statistically significant two-way interactions in SHIP-LEGEND and SHIP-Trend-0 on the lifetime MDD, as shown in Table 3.

#### Three-way interaction effects on current depressive symptoms using rs4588 as a proxy for 25(OH)D serum levels

The regressions with primary effects, two-way and three-way interactions on BDI-II were calculated in SHIP-LEGEND, SHIP-TREND-0, and the combined data set. In addition to the significant primary effect of childhood abuse (SHIP-LEGEND: *β* = 3.60 (2.55 – 4.64 95% CI), *p* = 1.62e-11; SHIP-TREND-0: *β* = 3.21 (2.55 – 3.87 95% CI), *p* < 2.2e-16; and combined: *β* = 3.38 (2.80 – 4.00 95% CI), *p* < 2.2e-16), no other significant primary or interaction effect with a *p*-value < 0.05 was found for current depressive symptoms, as shown in Supplementary Table S2.

## Discussion

This study investigated the moderation effect of vitamin D on the gene × environment interaction between the 5-HTTLPR polymorphism of the SLC6A4 gene and childhood abuse. We could demonstrate significant moderating effects of 25(OH)D or rs4588 in two-way interactions of childhood abuse and 5-HTTLPR on current or lifetime MDD, respectively. A possible explanation of this effect could be provided by the biological pathway of the TPH2 synthesis stimulation by vitamin D, which consequently increases the synthesis of serotonin in the brain. In our analyses, the S-allele-carrying genotypes were associated with the highest burden of depressive symptoms in subjects with both childhood abuse and low 25(OH)D serum levels. This finding is consistent with classical concepts of the role of serotonin in depressive disorders^2,19,20^. Under chronic stress and traumatization (childhood trauma), the brain´s homeostasis might be under constant challenge. limiting its adaptability to further stressful conditions^8^. The S-allele of the 5-HTTLPR may represent a risk factor for serotonin depletion as the synthesis of the presynaptic reuptake transporter is reduced compared to the L-allele^39^. Low 25(OH)D serum levels may lead to lower expression rates of the rate-limiting enzyme in serotonin synthesis, thus further increasing the presynaptic shortcoming of available serotonin, which could trigger the higher risk of depressive symptoms. The first part of our analysis clearly supports vitamin D´s role in serotonin-dependent mechanisms by moderating the 5-HTTLPR-childhood abuse interaction. Our finding that low 25(OH)D serum levels influence current depressive symptoms but not lifetime MDD is consistent with results from Almeida et al*.*^16^ and Milaneschi et al*.*^11^. While Almeida et al*.* showed that vitamin D deficiency was not associated with either past or future depression but only with current depressive symptoms^16^, Milaneschi et al*.* reported that vitamin D deficiency is not significantly associated with remitted depression^11^.

In the independent sample SHIP-LEGEND, we used SNP rs4588 as a proxy for the 25(OH)D serum levels. We chose this approach for an independent validation, taking advantage of the rather small (1%) but robust SNP effect on the 25(OH)D serum levels. Consistent with our hypothesis, we discovered a three-way interaction of rs4588, 5-HTTLPR, and childhood abuse on the risk of lifetime MDD in SHIP-LEGEND, and, as a replication, also in SHIP-TREND-0. Interestingly, a strong moderating effect of the 5-HTTLPR genotypes on the effects of the rs4588 genotypes in abused subjects was observed: the effects of a marked elevation in lifetime MDD risk were carried by the CC genotype (rs4588) in the SL/SS genotypes of the 5-HTTLPR. The SNP rs4588 represents a coding exon variant (THR- > LYS). The CC genotype of the vitamin D binding protein represents the high affinity genotype compared to AA. It leads to an increased binding of vitamin D to the binding protein (DBP), which resulted in higher 25(OH)D serum levels as measured in our assay. At first sight, this result seems to contradict the findings on 25(OH)D serum levels in SHIP-TREND-0 because here, a 25(OH)D deficiency was the risk for high BDI-II scores in the abused SL/SS group. However, the exact mechanisms of vitamin D metabolism with regard to the vitamin D binding protein remain to be clarified. The bioavailability of 25(OH)D as measured with our assay is probably lower in the high affinity CC genotype group because 25(OH)D is more tightly bound to DBP. The question about how to assess a disease-relevant vitamin D status is still partially unsolved^38^. The current golden standard, as performed in this study, is to measure the 25(OH)D in blood. However, new methods are available, such as assessing the vitamin D metabolite ratio (VMR), the ratio between 25(OH)D and 24,25-dihydroxy vitamin D, measuring only bioavailable 25(OH)D not bound to DBP, or measuring only free 25(OH)D, i.e., the circulating 25(OH)D bound to neither DBP nor albumin.

The risk status of the LL-genotype in the abused subjects was represented by the CA/AA low affinity group. Because the genotyped effects of rs4588 were the opposite (flipped) between the SL/SS and LL-genotypes, the interaction effects were rather strong and were successfully replicated in SHIP-TREND-0.

For dichotomous outcomes such as in those of MDD, another possible way to calculate the interaction risks is through the RERI (relative excess risk due to interaction)^40^. However, this method is not available for three-way interactions and therefore could not be applied in this paper.

One limitation to our study is that we did not analyse the different vitamin D metabolites and their varying bioavailability. Nevertheless, our analyses revealed a significant moderating effect of 25(OH)D as assessed by the gold standard measurement protocol for vitamin D. Our threshold for vitamin D deficiency with 17.2 ng/ml is close to but slightly lower than the corresponding medical threshold of 20 ng/ml. Since the medical threshold is not motivated by strong evidence for this exact value, we use the statistical threshold of 17.2 ng/ml here. Furthermore, the variation in rs4588 and the associated binding affinity of DBP accounts only for 1% of the serum levels of 25(OH)D compared to a seasonality contribution of 16–20%. Therefore, the precise investigation of the physiological effects of the rs4588 are challenging. However, our results still highlight its relevance to serotonin-mediated pathways. Last, we note that the retrospective measure of childhood abuse, which are inherent to our cohort-based study approach, may pose a certain limitation for the validity of our study^41^.

It is a strength of our study that we investigated two independent, highly characterized general population samples. We were thus able to adjust the analyses for major confounders of 25(OH)D serum levels, including the BMI, physical activity, smoking, and seasonality which may also influence mood and depressive symptoms.

We have added important new evidence to shed light on the putative physiological and pathophysiological roles of 5-HTTLPR and the serotonin system in subjects exposed to stressful life events and abuse in childhood. Our results may add to the discussion of the replicability of the classical two-way gene-environment interaction of the 5-HTTLPR and childhood trauma stimulated by recent papers by Border and Culverhouse^3,7^. In fact, it is important to note that this classical two-way interaction did not become significant in our sample and that only the introduction of the 25(OH)D serum level or the rs4588 genotypes uncovered underlying 5-HTTLPR-dependent effects on depressive symptoms and MDD. Thus, the 5-HTTLPR might exert its differential effects on mood and behaviour only under various threats to the serotonin homeostasis. This interpretation is consistent with our previous findings on relevant three-way interactions of the 5-HTTLPR and childhood trauma with the rs6265 of the BDNF gene^20^, the TPH2 gene^21^, and additional adult traumatization^19^.

While we may conclude in this paper that vitamin D moderates the interaction between 5-HTTLPR and abuse on depression, one might, of course, from a statistical point of view, also argue that 5-HTTLPR moderates the interaction between vitamin D and abuse on depression, since the two-way interaction between childhood abuse and vitamin D is significant while the interaction between 5-HTTLPR and childhood abuse is not. In fact, we have investigated three factors that interact with each other over long developmental periods and to date it has not been possible to determine on the underlying time sequence of the pathophysiological events. Our findings on the role of rs4588 also point to a model in which the vitamin D metabolism impacts vulnerability to depressive disorders early on.

Moreover, the complex interaction discovered in our study might also explain the seemingly negative association of vitamin D supplementation with depressive symptoms^42^.

In light of our results, supplementation with vitamin D could possibly be used in targeted prevention programmes in subjects exposed to childhood abuse, for those carrying the SL- or SS-allele of the 5-HTTLPR and who display low 25(OH)D (below 17.2 ng/ml). Given our results and the possible implications, we strongly believe that this hypothesis should to be tested in future clinical trials.

## Supplementary Information

Supplementary Information.

## Data Availability

The data that support the findings of this study are available from Transferstelle für Daten- und Biomaterialienmanagement. Restrictions apply to the availability of these data, which were used under license for this study. The data are available at https://www.fvcm.med.uni-greifswald.de/dd_service/data_use_intro.php with the permission of Transferstelle für Daten- und Biomaterialienmanagement.

## References

[1] Sullivan PF, Neale MC, Kendler KS (2000). Genetic epidemiology of major depression: review and meta-analysis. Am. J. Psychiatry.

[2] Caspi A (2003). Influence of life stress on depression: moderation by a polymorphism in the 5-HTT gene. Science.

[3] Culverhouse RC (2018). Collaborative meta-analysis finds no evidence of a strong interaction between stress and 5-HTTLPR genotype contributing to the development of depression. Mole. Psychiatry.

[4] Karg K, Burmeister M, Shedden K, Sen S (2011). The serotonin transporter promoter variant (5-HTTLPR), stress, and depression meta-analysis revisited: evidence of genetic moderation. Arch. Gen. Psychiatry.

[5] Munafò MR, Durrant C, Lewis G, Flint J (2009). Gene × Environment Interactions at the Serotonin Transporter Locus. Biol. Psychiat..

[6] Risch N (2009). Interaction between the serotonin transporter gene (5-HTTLPR), stressful life events, and risk of depression: a meta-analysis. JAMA.

[7] Border R (2019). No support for historical candidate gene or candidate gene-by-interaction hypotheses for major depression across multiple large samples. AJP.

[8] Cathomas F, Murrough JW, Nestler EJ, Han M-H, Russo SJ (2019). Neurobiology of Resilience: Interface Between Mind and Body. Biol. Psychiat..

[9] Anglin RES, Samaan Z, Walter SD, McDonald SD (2013). Vitamin D deficiency and depression in adults: systematic review and meta-analysis. Br. J. Psychiatry.

[10] Eyles DW, Burne THJ, McGrath JJ (2013). Vitamin D, effects on brain development, adult brain function and the links between low levels of vitamin D and neuropsychiatric disease. Front. Neuroendocrinol..

[11] Milaneschi Y (2014). The association between low vitamin D and depressive disorders. Mole. Psychiatry.

[12] Kaneko I (2015). 1,25-Dihydroxyvitamin D regulates expression of the tryptophan hydroxylase 2 and leptin genes: implication for behavioral influences of vitamin D. FASEB J..

[13] Goltz A (2018). Association of brain-derived neurotrophic factor and vitamin D with depression and obesity: a population-based study. Neuropsychobiology.

[14] Jääskeläinen T (2015). Higher serum 25-hydroxyvitamin D concentrations are related to a reduced risk of depression. Br. J. Nutr..

[15] Parker GB, Brotchie H, Graham RK (2017). Vitamin D and depression. J. Affect. Disord..

[16] Almeida OP, Hankey GJ, Yeap BB, Golledge J, Flicker L (2015). Vitamin D concentration and its association with past, current and future depression in older men: the health in men study. Maturitas.

[17] Schulz A (2014). The impact of childhood trauma on depression: Does resilience matter? Population-based results from the study of health in pomerania. J. Psychosom. Res..

[18] Gilbert R (2009). Burden and consequences of child maltreatment in high-income countries. Lancet (London, England).

[19] Grabe HJ (2012). Moderation of adult depression by the serotonin transporter promoter variant (5-HTTLPR), childhood abuse and adult traumatic events in a general population sample. Am. J. Med. Genet. Part B Neuropsychiatr. Genet.

[20] Grabe HJ (2012). Genetic epistasis between the brain-derived neurotrophic factor Val66Met polymorphism and the 5-HTT promoter polymorphism moderates the susceptibility to depressive disorders after childhood abuse. Prog. Neuropsychopharmacol. Biol. Psychiatry.

[21] Van der Auwera S (2014). Interaction among childhood trauma and functional polymorphisms in the serotonin pathway moderate the risk of depressive disorders. Eur. Arch. Psychiatry Clin. Neurosci..

[22] Sinotte M, Diorio C, Bérubé S, Pollak M, Brisson J (2009). Genetic polymorphisms of the vitamin D binding protein and plasma concentrations of 25-hydroxyvitamin D in premenopausal women. Am. J. Clin. Nutr.

[23] O’Brien KM (2018). Genome-wide association study of serum 25-hydroxyvitamin D in US women. Frontiers Genet..

[24] McGrath JJ, Saha S, Burne THJ, Eyles DW (2010). A systematic review of the association between common single nucleotide polymorphisms and 25-hydroxyvitamin D concentrations. J. Steroid Biochem. Mole. Biol.

[25] Grabe HJ (2005). Mental and physical distress is modulated by a polymorphism in the 5-HT transporter gene interacting with social stressors and chronic disease burden. Mole. Psychiatry.

[26] John U (2001). Study of health in pomerania (ship): a health examination survey in an east German region: objectives and design. Sozial- und Präventivmedizin SPM.

[27] Völzke H (2011). Cohort profile: the study of health in Pomerania. Int. J. Epidemiol..

[28] Wittchen H-U, Lachner G, Wunderlich U, Pfister H (1998). Test-retest reliability of the computerized DSM-IV version of the munich-composite international diagnostic interview (M-CIDI). Soc. Psychiatry Psychiatr. Epidemiol..

[29] Beck A, Steer R (1987). Beck Depression Inventory -Manual.

[30] Kroenke K, Spitzer RL, Williams JB (2001). The PHQ-9: validity of a brief depression severity measure. J Gen Intern Med.

[31] Wahl I (2014). Standardization of depression measurement: a common metric was developed for 11 self-report depression measures. J. Clin. Epidemiol..

[32] Bernstein DP (2003). Development and validation of a brief screening version of the childhood trauma questionnaire. Child Abuse Negl..

[33] Wingenfeld K (2010). Die deutsche Version des Childhood Trauma Questionnaire (CTQ): Erste Befunde zu den psychometrischen Kennwerten. PPmP.

[34] Hu X-Z (2006). Serotonin transporter promoter gain-of-function genotypes are linked to obsessive-compulsive disorder. Am. J. Hum. Genet.

[35] Perroud N (2010). Rare genotype combination of the serotonin transporter gene associated with treatment response in severe personality disorder. Am. J. Med. Genet. Part B Neuropsychiatric Genet..

[36] Harrell F (2001). Regression Modeling Strategies with Applications to Linear Models, Logistic Regression, and Survival Analysis.

[37] R Core Team. *R: A language and environment for statistical computing*. (R Foundation for Statistical Computing, 2019).

[38] Herrmann M, Farrell C-JL, Pusceddu I, Fabregat-Cabello N, Cavalier E (2017). Assessment of vitamin D status—a changing landscape. Clin. Chem. Lab. Med. (CCLM).

[39] Neumeister A (2006). Differential effects of 5-HTTLPR genotypes on the behavioral and neural responses to tryptophan depletion in patients with major depression and controls. Arch. Gen. Psychiatry.

[40] Grabe HJ (2009). Serotonin transporter gene (slc6a4) promoter polymorphisms and the susceptibility to posttraumatic stress disorder in the general population. AJP.

[41] Baldwin JR, Reuben A, Newbury JB, Danese A (2019). Agreement between prospective and retrospective measures of childhood maltreatment: a systematic review and meta-analysis. JAMA Psychiatry.

[42] Li G (2014). Efficacy of vitamin d supplementation in depression in adults: a systematic review. J. Clin. Endocrinol. Metab.

