# Supplementary material for: Vitamin D moderates the interaction between 5-HTTLPR and childhood abuse in depressive disorders
Source: Sci Rep. 2020 Dec 28;10:22394. doi: 10.1038/s41598-020-79388-7 (PMC7769965; doi:10.1038/s41598-020-79388-7)
Supplement: Supplementary file 1 — Supplementary Information. [file 41598_2020_79388_MOESM1_ESM.pdf]

Supplementary information for:

Vitamin D moderates the interaction between 5-HTTLPR and childhood abuse in depressive disorders

Sarah Bonk[1], Johannes Hertel, PhD[1,2], Helena U. Zacharias, PhD[1], Jan Terock, M.D.[1, 3], Deborah Janowitz, M.D.[1], Georg Homuth, PhD[4], Matthias Nauck, M.D.[5,6], Henry Völzke, M.D.[6,7], Henriette Meyer zu Schwabedissen, M.D.[8], Sandra Van der Auwera, PhD[1,9#], Hans Jörgen Grabe, M.D.[1,9#]

[1]Department of Psychiatry and Psychotherapy, University Medicine Greifswald, Germany
[2] Molecular Systems Physiology group, National University of Ireland, Galway, Ireland
[3]Department of Psychiatry and Psychotherapy, HELIOS Klinikum Stralsund, Germany
[4]Interfaculty Institute for Genetics and Functional Genomics, University Medicine Greifswald, Germany
[5]Institute of Clinical Chemistry and Laboratory Medicine, University Medicine Greifswald, Germany
[6]DZHK (German Centre for Cardiovascular Research), Partner Site Greifswald, University Medicine, Greifswald, Germany
[7]Institute for Community Medicine, University Medicine Greifswald, Greifswald, Germany
[8]Biopharmacy, Department Pharmaceutical Sciences, University of Basel, Switzerland
[9] German Center for Neurodegenerative Diseases DZNE, Site Rostock/ Greifswald, Germany
[#]These authors contributed equally.

Email: sarah.bonk@med.uni-greifswald.de

METHODS

Sampling and phenotyping methods

SAMPLE AND SAMPLE RECRUITMENT

Data from the Study of Health in Pomerania (SHIP) were used (Grabe et al., 2005; John et al., 2001; Völzke et al., 2011). The target population comprised

adult German residents in northeastern Germany living in 3 cities and 29 communities, with a total population of 212,157. A two-stage stratified cluster sample of adults aged 20 to 79 years (baseline) was drawn at random from local population registration files in which every resident has to be included by law. Among the net sample (without migrated or deceased persons) of 6,267 eligible subjects, 4,308 Caucasian subjects participated at baseline (SHIP-0) between 1997 and 2001. Follow-ups, named SHIP 1 to 3, have been conducted 5, 11, and 17 years after the baseline.

From June 2007 until August 2010, the ''Life-Events and Gene-Environment Interaction in Depression (SHIP-LEGEND)'' study was carried out (Völzke et al., 2011). Until the beginning of SHIP-LEGEND, 639 participants from the baseline sample SHIP-0 were either deceased (*n*=383) or refused further participation (*n*=256). Thus, 3,669 participants were invited to take part in the SHIP-LEGEND study, from which 2,400 participated.

In 2008, a new independent sample (SHIP–TREND-0) with 4,420 subjects from the same area was drawn and examinations similar to SHIP-0 were undertaken.

We excluded subjects from both cohorts with missing information on childhood trauma, genetic polymorphisms (5-HTTLPR, rs25531 or rs4588), MDD, or BDI-II/PHQ-9. The remaining sample comprised *n*=1,997 subjects of SHIP-LEGEND and *n*=2,939 subjects of SHIP-TREND-0. For the interaction analyses based on measured vitamin D (25(OH)D) *n*=2,901 subjects were available in SHIP-TREND-0.

PHENOTYPE MEASURES

In SHIP-LEGEND and SHIP-TREND-0, a diagnostic interview for mental disorders was performed based on Diagnostic and Statistical Manual for Mental Disorders (IV edition) diagnostic criteria (Völzke et al., 2011; Wittchen, Lachner, Wunderlich, & Pfister, 1998). Additional psychometric assessments included the Beck depression inventory (BDI-II, SHIP-LEGEND), patient health questionnaire (PHQ-9, SHIP-TREND-0) and childhood trauma questionnaire (CTQ, SHIP-LEGEND and SHIP-TREND-0). The BDI-II measures current depressive symptoms with high reliability and validity using a 21-item self-

report questionnaire (Beck & Steer, 1987). PHQ-9, which is used in SHIP-TREND-0, is a 9-item self-report questionnaire also with high reliability and validity (Kroenke, Spitzer, & Williams, 2001).
The PHQ-9 score was transformed into the BDI-II according to Wahl et al. (Wahl et al., 2014) to create one common variable on depressive symptoms for both cohorts.
Our PHQ-9 scores of SHIP-TREND-0 and the transformed BDI-II values agreed well with a correlation > 0.9.
CTQ was used for self-report of childhood maltreatment including emotional, physical and sexual abuse (Bernstein et al., 2003; Schulz et al., 2014; Wingenfeld et al., 2010). It comprises 34 items rated on a five-point Likert scale with higher scores indicating more self-rated exposure to traumatic events. In addition to dimensional scoring procedure, the following threshold scores to determine the severity of abuse were used: none = 0, mild = 1, moderate = 2 and severe to extreme = 3. To investigate the role of an increasing severity of childhood abuse in G x E interactions, we generated a dichotomized variable of overall abuse. A subject was rated as positive for overall abuse when at least in one of the abuse sub-dimensions a severity score of at least mild was reported.

VITAMIN D MEASUREMENT

Venous blood samples were taken in SHIP-TREND-0 from the cubital vein of the participants in the supine position. The samples were taken throughout the year and stored at -80°C. Serum 25(OH)D concentrations were determined on the IDS-iSYS Multi-Discipline Automated Analyser (Immunodiagnostic Systems Limited, Frankfurt am Main, Germany). For quality control purposes, three concentrations of control material were measured. In SHIP-TREND-0 the coefficients of variation in control material were 11.6% at low concentrations, 9.1% at medium concentrations, and 10.6% at high concentrations, respectively.
For the regression analysis, the continuous 25(OH)D serum levels were divided into four quantiles, with the quartile containing the lowest

25(OH)D serum values considered as risk group against the remaining sample.

Genetic methods

GENOTYPING OF THE 5-HTTLPR

The SLC6A4 gene harbors a variable number tandem repeat (VNTR) polymorphism in the transcription control region of the gene that is located approximately 1 kb upstream of the transcription initiation site. This area has been associated with differential expression of the transporter (rs4795541) (Heils et al., 1995). Both variants (Short, Long) differ by a 43-bp insertion/deletion ("biallelic" 5-HTTLPR). Within the inserted fragment, an additional common single nucleotide polymorphism (SNP) occurs (rs25531) and has been reported to further affect the transcriptional activity of the *SLC6A4* promoter by the genotype-dependent generation of an AP2 transcription factor binding site in the rs25531 G-allele (Hu et al., 2006). This suggests that 5-HTTLPR is triallelic with S, L_A and L_G —alleles.
We developed a restriction fragment length polymorphism (RFLP) method that allows for determination of both variants (S/L; rs25531) within one assay. The 5-HTTLPR region was PCR amplified using the oligonucleotide primers SLC6A4_SE (5'-CTCCTAGGATCGCTCCTGCATC-3') and SLC6A4_AS (5'-GGACCGCAAGGTGG-GCGGGAGGCTTGGAG-3'), resulting in amplicons of 294 bp (S-variant) and 337 bp (L-variant). The restriction enzyme *BcnI* (Fermentas) digested the rs25531 variant differentially, in addition to two constitutive restriction sites in the amplicon. This resulted in the following fragments: S-allele, 200, 61, 33 bp; L_A -allele, 243, 61, 33 bp; and L_G -allele, 70, 173, 61, 33 bp. The detection of fragments of 173, 200, or 243 bp in 4 % agarose gels allowed for allocation to the respective alleles. Representative samples of different genotypes were further verified by sequencing of the amplicons. Based on previous reports on gene expression, we classified the genotypes into three functional ''triallelic'' genotypes: L_AL_A = LL; L_GL_A or SL_A = SL; L_GL_G or L_GS or SS = SS (Hu et al., 2006). However, recently, the functional relevance of rs25531 has been called into question (Perroud et al., 2010). Still, we report the results of the three-way interaction for this triallelic

5-HTTLPR. While SS and SL are considered separately in the descriptive statistics, they are grouped together for the regression (Grabe et al., 2005).

GENOTYPING OF rs4588

The SHIP-0 sample (*n*=4,070) was genotyped using the Affymetrix Human SNP Array 6.0. The overall genotyping efficiency was 98.55%. Genotyping in a subset of the SHIP-TREND-0 study (*n*=986) was performed using the Illumina HumanOmni 2.5-Quad. The final sample call rate was 99.51%. The remaining SHIP-TREND-0 sample (*n*=3,133) was genotyped using the Illumina GSA-24. Arrays with a genotyping call rate <94% were removed. For further details, see (Völzke et al., 2011).

Imputation of genotypes was performed using the HRCv1.1 reference panel and the Eagle and minimac3 software implemented in the Michigan Imputation Server for pre-phasing and imputation, respectively. SNPs with a Hardy-Weinberg-Equilibrium *p*-value <0.0001, a call rate <0.95, and a MAF <1% were removed before imputation.

The SNP was imputed with imputation quality >0.99 in all three batches. The frequencies of the genotypes in the two samples were AA:161, AC:838, CC:998 in SHIP-LEGEND and AA:239, AC:1,273, CC:1,427 in SHIP-TREND-0. Due to the low minor allele frequency and the small number of homozygote AA carriers we grouped AA/AC versus CC in the regression analyses.

SEASONAL EFFECT ON VITAMIN D

The seasonal 25(OH)D variations are depicted in Fig. S1 with the subjects of SHIP-TREND-0 divided into two groups: no depression BDI<11 represented by a blue dashed line, and at least light depression BDI ≥ 11represented by a red solid line. Subjects with elevated depressive symptoms had lower 25(OH)D serum levels. Season explained 22.2% of the variance of vitamin D.

SUPPLEMENTARY FIGURES

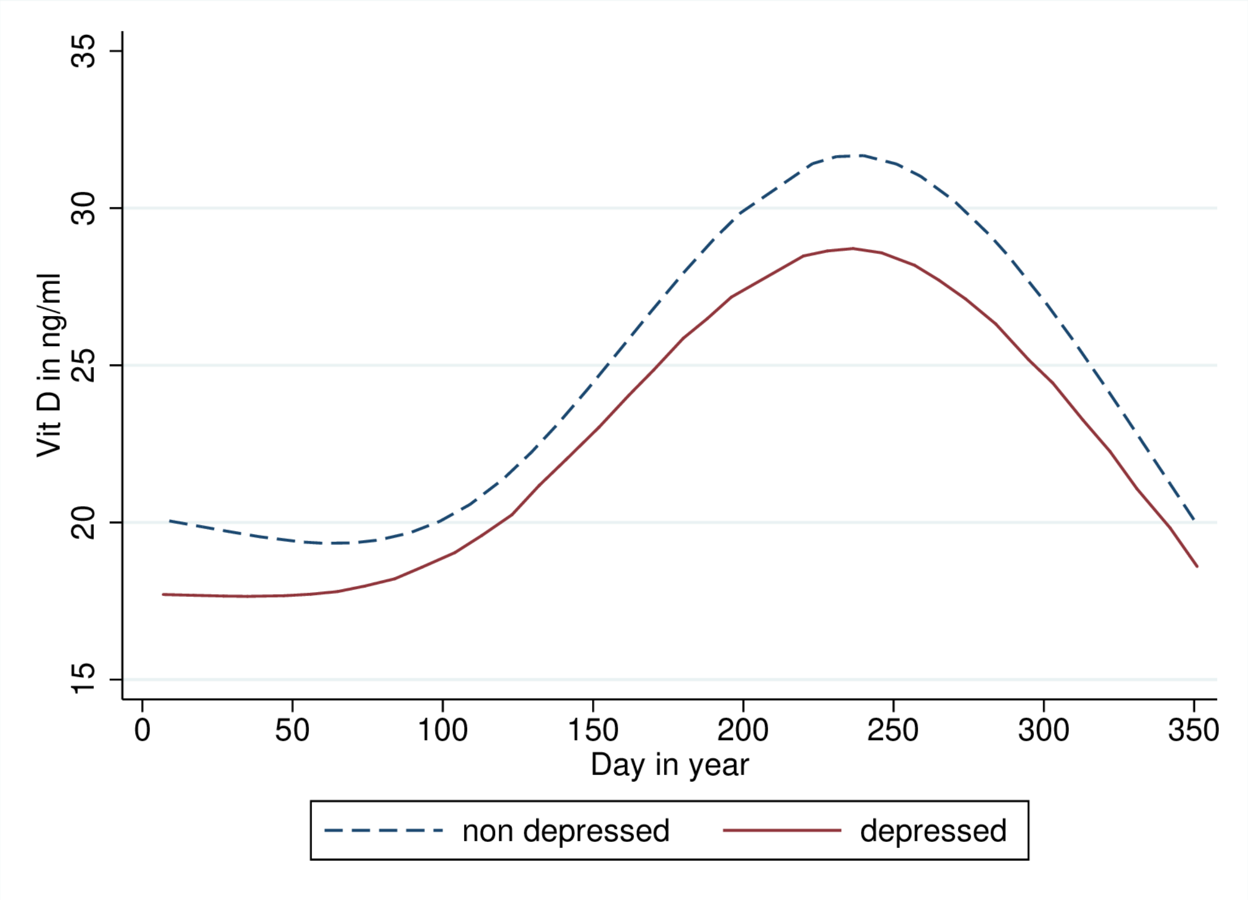

Fig. S1: Seasonal variation of Vitamin D in ng/ml over the year in non-depressed and at least mildly depressed subjects in SHIP-TREND-0.

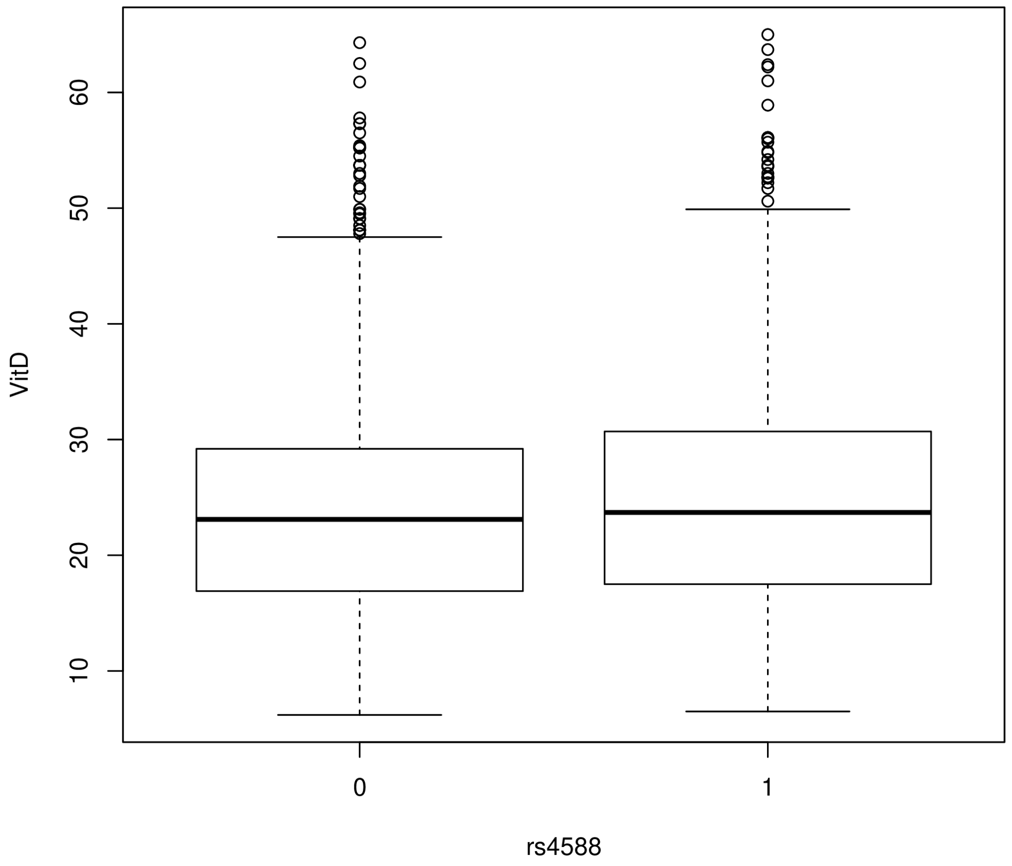

Fig. S2: Boxplot of 25-Hydroxyvitamin D serum values in ng/ml for different rs4588 genotypes (0=CC, 1=CA/AA) in SHIP-TREND-0.

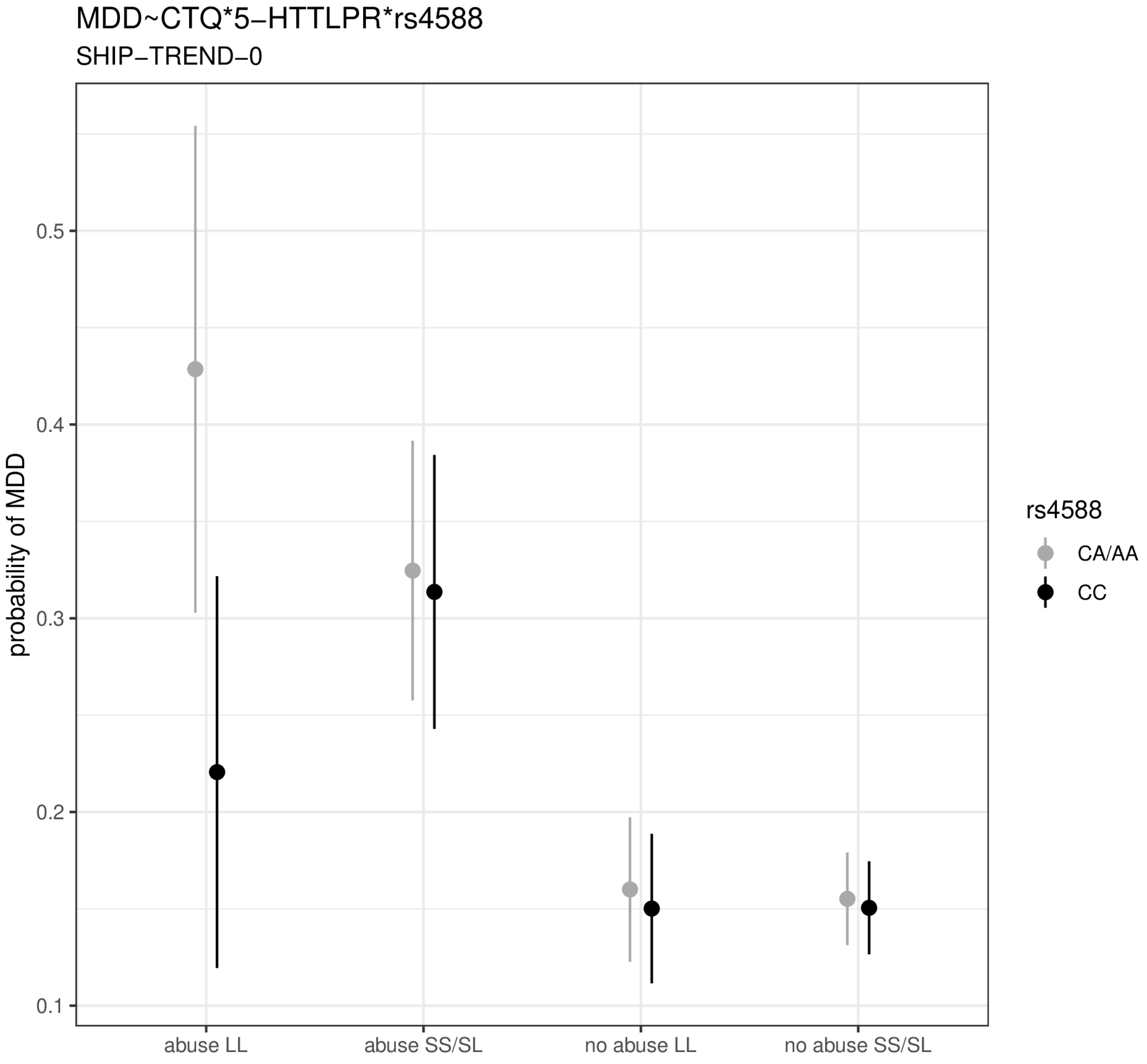

Fig. S3: Mean and confidence intervals of probabilities of lifetime depression (MDD) for different subgroups of the three-way interaction childhood trauma questionnaire (CTQ, abuse vs. no abuse), 5-HTTLPR (LL vs. SS/SL), and rs4588(CC vs. CA/AA) in SHIP-TREND-0.

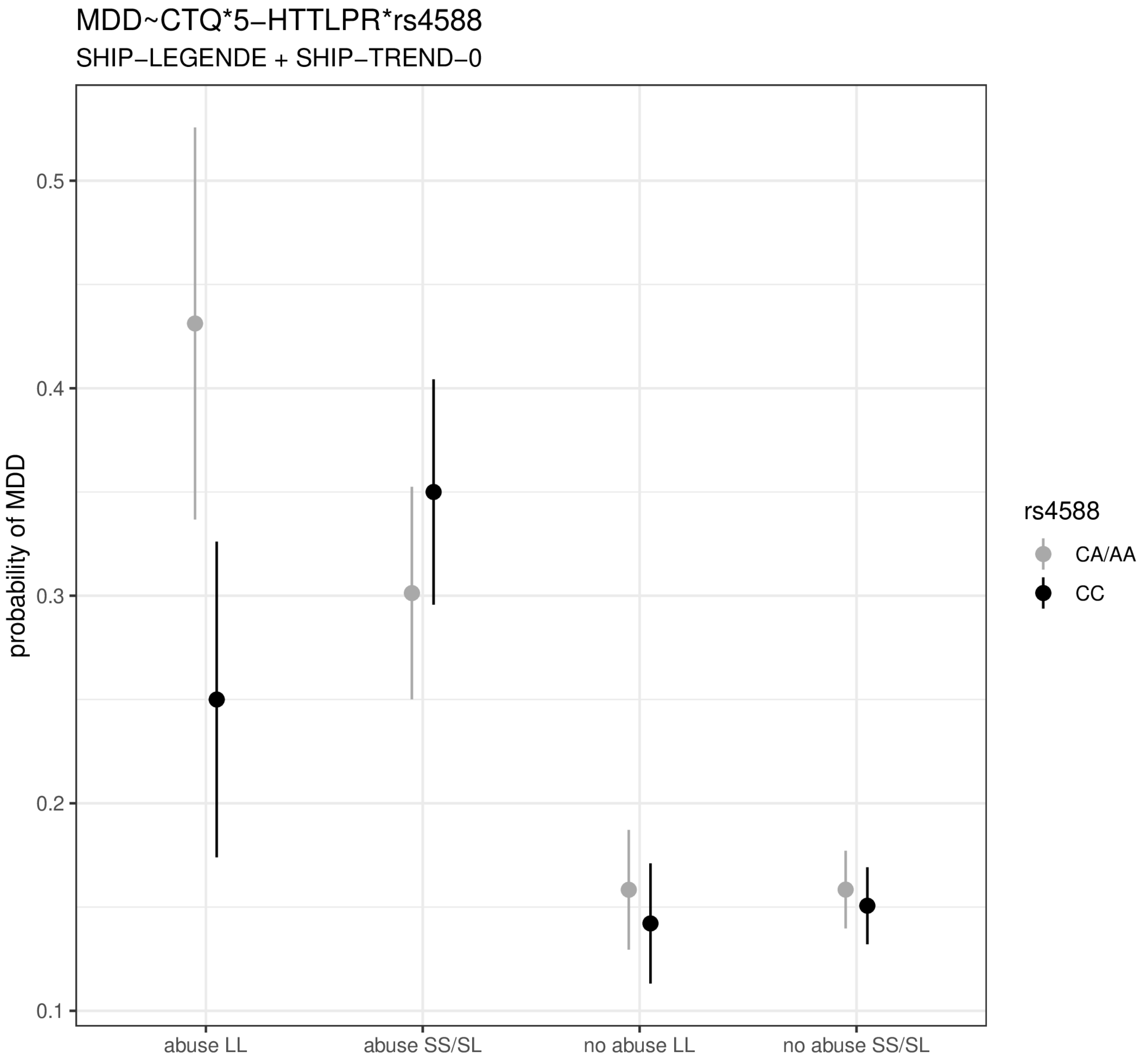

Fig. S4: Mean and confidence intervals of probabilities of lifetime depression (MDD) for different subgroups of the three-way interaction childhood trauma questionnaire (CTQ, abuse vs. no abuse), 5-HTTLPR (LL vs. SL/SS), and rs4588(CC vs. CA/AA) in the combined sample (SHIP-LEGEND + SHIP-TREND-0).

SUPPLEMENTARY TABLES

Outcome: MDD	**SHIP-TREND-0**	
	OR (95% CI)	*p*-value
**Primary effect**		
Abuse	2.50 (1.99 - 3.13)	2.12e-15
5-HTTLPR	1.03 (0.83 - 1.27)	0.803
25(OH)D	1.25 (0.99 - 1.57)	0.063
**Two-way interaction**		
Abuse*5-HTTLPR	0.99 (0.60 – 1.64)	0.976
Abuse*25(OH)D	1.37 (0.83 – 2.27)	0.217
5-HTTLPR*25(OH)D	1.29 (0.80 – 2.07)	0.289
**Three-way interaction**		
Abuse*5-HTTLPR*25(OH)D	0.97 (0.31 – 2.97)	0.951

Tab. S1: Logistic regression of MDD and different primary effects with robust estimates adjusted for age and sex. For the effects including the 25OHD, the regression has additionally been adjusted for the body mass index, physical activity, smoking and season in SHIP-TREND-0. Abbreviations: OR, odds ratio ; CI, confidence interval.

Outcome: BDI-II	**SHIP-LEGEND**		**SHIP-TREND-0**		**Combined**	
	β(95% CI) coefficient	*p*-value	β(95% CI) coefficient	*p*-value	β(95% CI) coefficient	*p*-value
**Primary effect**						
Abuse	3.60 (2.55 – 4.64)	1.62e-11	3.21 (2.55 – 3.87)	<2.2e-16	3.38 (2.80 – 3.96)	<2.2e-16
5-HTTLPR	-0.55 (-1.23 – 0.13)	0.115	-0.04 (-0.49 – 0.40)	0.85	-0.23 (-0.65 – 0.12)	0.173
rs4588	-0.43 (-1.08 – 0.22)	0.33	-0.10 (-0.49 – 0.30)	0.627	-0.23 (-0.58 – 0.13)	0.208
**Two-way interaction**						
Abuse*5-HTTLPR	0.97 (-1.26 – 3.21)	0.394	0.61 (-0.96 – 2.17)	0.447	0.76 (-0.55 – 2.07)	0.255
Abuse*rs4588	1.70 (-0.40 – 3.79)	0.112	-0.75 (-2.06 - 0.56)	0.260	0.25 (-0.92 – 1.41)	0.680
5-HTTLPR*rs4588	-0.62 (-1.98 – 0.74)	0.371	-0.04 (-0.93 – 0.85)	0.929	-0.33 (-1.10 – 0.43)	0.395
**Three-way interaction**						
Abuse*5-HTTLPR*rs4588	-3.43 (-7.84 – 0.98)	0.128	-1.29 (-4.42 – 1.83)	0.417	-2.13 (-4.74 – 0.48)	0.110

Tab. S2: Linear regression on BDI-II scores and different primary effects with robust estimates adjusted for age, sex and study cohort in SHIP-LEGEND, SHIP-TREND-0 and the combined sample (SHIP-LEGEND + SHIP-TREND-0). Abbreviation: CI, confidence interval.

Beck, A., & Steer, R. (1987). *Beck Depression Inventory -Manual*. San Antino: The Physiological Corporation.

Bernstein, D. P., Stein, J. A., Newcomb, M. D., Walker, E., Pogge, D., Ahluvalia, T., … Zule, W. (2003). Development and validation of a brief screening version of the Childhood Trauma Questionnaire. *Child Abuse & Neglect*, *27*(2), 169–190. https://doi.org/10.1016/S0145-2134(02)00541-0

Grabe, H. J., Lange, M., Wolff, B., Völzke, H., Lucht, M., Freyberger, H. J., … Cascorbi, I. (2005). Mental and physical distress is modulated by a polymorphism in the 5-HT transporter gene interacting with social stressors and chronic disease burden. *Molecular Psychiatry*, *10*(2), 220–224. https://doi.org/10.1038/sj.mp.4001555

Heils, A., Teufel, A., Petri, S., Seemann, M., Bengel, D., Balling, U., … Lesch, K.-P. (1995). Functional promoter and polyadenylation site mapping of the human serotonin (5-HT) transporter gene. *Journal of Neural Transmission*, *102*(3), 247–254. https://doi.org/10.1007/BF01281159

Hu, X.-Z., Lipsky, R. H., Zhu, G., Akhtar, L. A., Taubman, J., Greenberg, B. D., … Goldman, D. (2006). Serotonin Transporter Promoter Gain-of-Function Genotypes Are Linked to Obsessive-Compulsive Disorder. *The American Journal of Human Genetics*, *78*(5), 815–826. https://doi.org/10.1086/503850

John, U., Hensel, E., Lüdemann, J., Piek, M., Sauer, S., Adam, C., … Kessler, C. (2001). Study of Health in Pomerania (SHIP): A health examination survey in an east German region: Objectives and design. *Sozial- Und Präventivmedizin SPM*, *46*(3), 186–194. https://doi.org/10.1007/BF01324255

Kroenke, K., Spitzer, R. L., & Williams, J. B. (2001). The PHQ-9: Validity of a brief depression severity measure. *Journal of General Internal Medicine*, *16*(9), 606–613.

Perroud, N., Salzmann, A., Saiz, P. A., Baca-Garcia, E., Sarchiapone, M., Garcia-Portilla, M. P., … and European Research Consortium for Suicide (EURECA). (2010). Rare genotype combination of the serotonin transporter gene associated with treatment response in severe

personality disorder. *American Journal of Medical Genetics Part B: Neuropsychiatric Genetics*, *153B*(8), 1494–1497. https://doi.org/10.1002/ajmg.b.31118

Schulz, A., Becker, M., Van der Auwera, S., Barnow, S., Appel, K., Mahler, J., … Grabe, H. J. (2014). The impact of childhood trauma on depression: Does resilience matter? Population-based results from the Study of Health in Pomerania. *Journal of Psychosomatic Research*, *77*(2), 97–103. https://doi.org/10.1016/j.jpsychores.2014.06.008

Völzke, H., Alte, D., Schmidt, C. O., Radke, D., Lorbeer, R., Friedrich, N., … Hoffmann, W. (2011). Cohort Profile: The Study of Health in Pomerania. *International Journal of Epidemiology*, *40*(2), 294–307. https://doi.org/10.1093/ije/dyp394

Wahl, I., Löwe, B., Bjorner, J. B., Fischer, F., Langs, G., Voderholzer, U., … Rose, M. (2014). Standardization of depression measurement: A common metric was developed for 11 self-report depression measures. *Journal of Clinical Epidemiology*, *67*(1), 73–86. https://doi.org/10.1016/j.jclinepi.2013.04.019

Wingenfeld, K., Spitzer, C., Mensebach, C., Grabe, H., Hill, A., Gast, U., … Driessen, M. (2010). Die deutsche Version des Childhood Trauma Questionnaire (CTQ): Erste Befunde zu den psychometrischen Kennwerten. *PPmP - Psychotherapie · Psychosomatik · Medizinische Psychologie*, *60*(11), 442–450. https://doi.org/10.1055/s-0030-1247564

Wittchen, H.-U., Lachner, G., Wunderlich, U., & Pfister, H. (1998). Test-retest reliability of the computerized DSM-IV version of the Munich-Composite International Diagnostic Interview (M-CIDI). *Social Psychiatry and Psychiatric Epidemiology*, *33*(11), 568–578. https://doi.org/10.1007/s001270050095
